# Differential susceptibility to obesity between male, female and ovariectomized female mice

**DOI:** 10.1186/1475-2891-8-11

**Published:** 2009-02-17

**Authors:** Jina Hong, Renee E Stubbins, Rebekah R Smith, Alison E Harvey, Nomelí P Núñez

**Affiliations:** 1 Department of Nutritional Sciences, University of Texas at Austin, Austin, Texas, USA

## Abstract

**Background:**

The prevalence of obesity has increased dramatically. A direct comparison in the predisposition to obesity between males, premenopausal females, and postmenopausal females with various caloric intakes has not been made. To determine the effects of sex and ovarian hormones on the susceptibility to obesity, we conducted laboratory studies with mice. To eliminate confounders that can alter body weight gain, such as age and food consumption; we used mice with the same age and controlled the amount of calories they consumed.

**Methods:**

We determined sex-specific susceptibility to obesity between male, non-ovariectomized female, and ovariectomized female mice. To compare susceptibility to gaining body weight between males and females, animals from each sex were exposed to either a 30% calorie-restricted, low-fat (5% fat), or high-fat (35% fat) diet regimen. To establish the role of ovarian hormones in weight gain, the ovaries were surgically removed from additional female mice, and then were exposed to the diets described above. Percent body fat and percent lean mass in the mice were determined by dual energy x-ray absorptiometry (DEXA).

**Results:**

In all three diet categories, male mice had a greater propensity of gaining body weight than female mice. However, ovariectomy eliminated the protection of female mice to gaining weight; in fact, ovariectomized female mice mimicked male mice in their susceptibility to weight gain. In summary, results show that male mice are more likely to become obese than female mice and that the protection against obesity in female mice is eliminated by ovariectomy.

**Conclusion:**

Understanding metabolic differences between males and females may allow the discovery of better preventive and treatment strategies for diseases associated with body weight such as cancer and cardiovascular disease.

## Background

The prevalence of overweight and obesity has increased dramatically in the US population [1]. It is predicted that by 2030, 86% adults will be overweight or obese, and by 2048, all American adults will become overweight or obese [1]. The role gender plays in the susceptibility to obesity is not fully understood, specifically the role ovarian hormones play. Epidemiological studies that focus on determining the role gender has on the susceptibility to obesity have provided valuable information, showing that "women generally have a larger proportion of body mass as fat, and are more likely to deposit fat subcutaneously and on their lower extremities; men are more likely to deposit fat in the abdominal region" [2]. However, epidemiological studies have been limited by confounding factors (e.g., occupational differences between males and females) in determining the role gender and ovarian hormones play in the susceptibility to obesity. Confounding factors that can alter the effects of gender and ovarian hormones on the susceptibility to obesity include: 1) occupation differences between males and females (some jobs require more physical activity than others), 2) recreational physical activity differences between males and females, 3) differences in initial body composition at the beginning of the study between the sexes, 4) concomitant food intake pattern differences between males and females, and 5) reproductive differences (e.g., weight fluctuations during and after pregnancy). In animal studies, however, many of these confounding factors and others, such as age and genetics, can be controlled for; moreover, experimental animals can be closely monitored throughout the study to measure factors that affect body weight gain (e.g., initial body weight, diet, calorie intake, and physical activity). Review of the animal literature investigating the predisposition to obesity between males, females and ovarian hormones, revealed a lack of studies in this area. In fact, a direct comparison in the predisposition to obesity between males, females, and ovariectomized females on various diet regimens has not been made. We and others have shown that male mice have a higher susceptibility to become obese compared to female mice [3,4]. Moreover, we have shown that removal of ovarian hormones (surgical removal of the ovaries by ovariectomy) increased the susceptibility of female mice to become obese [5,6]. However, we have not simultaneously compared the predisposition to obesity between male, female and ovariectomized female mice receiving different diets. We conducted studies in mice to eliminate confounding factors that affect susceptibility to obesity, such as age, genetics, calorie intake and diet. Our results show that male mice are more susceptible to obesity than female mice, and that ovariectomy eliminates the protection against weight gain in female mice; in fact, ovariectomized female mice appear to mimic the male mice in their susceptibility to weight gain and percent body fat levels.

## Methods and procedures

At six weeks of age, C57BL/6 male, female and ovariectomized female mice (Charles River Laboratories) were randomized (15 per group) to receive one of three diet regimens: 30% calorie-restricted (CR), low-fat (5% fat) (LF), or high-fat (35% fat) (HF). A table with detailed diet formulations has been previously published [6]. Briefly, the CR diet contained 27% protein, 54% carbohydrate, and 6% fat; the LF diet contained 19.2% protein, 67.3% carbohydrate, and 4.3% fat; and the HF diet contained 26% protein, 26% carbohydrate, and 35% fat. The CR diet was modified so that the mice received 70% of the mean daily caloric consumption but 100% of the vitamins and minerals of the LF groups. Mice were singly housed, received their dietary treatments for 20 weeks, provided with their respective diets either *ad libitum *or calorie restricted, and kept on a 12-h light/dark cycle. Food consumption was recorded twice weekly and body weight weekly. Animal protocol was approved by the Institutional Animal Care and Use Committee at the University of Texas at Austin.

### Ovariectomy

Surgical removal of the ovaries is a well-characterized approach to mimic the postmenopausal state in mice [3]. In short, mice were anesthetized with Avertin. Hair was clipped over the surgical area and scrubbed with Betadine and ethanol swipe. A small midline incision (~1.0 cm) was made in the skin halfway between the middle of the back and the base of the tail, starting at the last rib. The skin was moved to one side, and a small incision was made through the peritoneal lining on each side. The entire ovary was removed with a single cut between the fallopian tube and the uterine horn. The skin was then closed with a surgical staple.

### Body composition

Body composition was determined using a GE Lunar Piximus Densitometer [3]. Briefly, at the end of the study, carcasses were stored at 80°C. To determine body fat levels frozen carcasses were thawed for 24 hours at 4°C. The thawed carcasses were placed on a tray lying face down with limbs and tail outstretched. Carcasses were weighed and scanned individually. A General Electric (GE)-supplied software (version 1.46) was used to exclude the heads from each mouse from the image area; then estimates of percent lean and fat were obtained directly from the DEXA instrument output.

### Statistical analyses

Two-way analysis of variance (ANOVA) was used to assess the effects of sex and diet within the various groups. We report the results of ANOVA and *a posteriori *comparison of the means using Tukey's Honestly Significant Difference procedure. To make comparisons within the groups, α was set to 0.05 (e.g. CR male vs. HF male; and between groups, CR male vs. CR female). Analysis was done in the final body weight and percent body fat of the mice

## Results

For twenty weeks, male, female and OVX female mice were exposed to either a CR, LF, or HF diet. Baseline body weight for all male mice was 20 ± 0.1 grams (± SE); values among the various groups were not significantly different (p > 0.05). Final body weight for CR male mice was 20 ± 0.2, LF 35 ± 0.8, and HF mice 46 ± 1.0. Baseline average body weight for all female mice was 18 ± 0.2 grams; there were no significant differences between groups. Final body weight for female groups was: CR 18 ± 0.3, LF 27 ± 0.7, and HF 32 ± 1.3. Baseline body weight for all OVX-female mice was 20 ± 0.2 grams; baseline weights were not significantly different among OVX mice. Final body weight for OVX-female groups was: CR 22 ± 0.4, LF 32 ± 1.0, and HF 47 ± 2.1.

**Figure 1:**
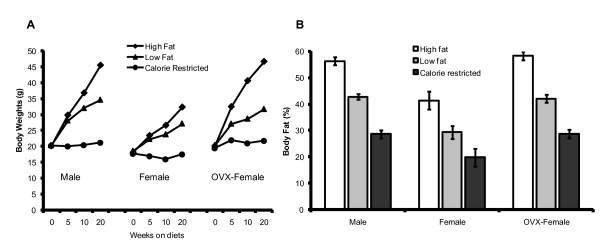
**A, shows the body weight profiles for male, female and OVX female C57BL/6 mice fed CR, LF, or HF diets for 20 weeks**. Male mice had a greater propensity to gain weight and become obese than female mice (p < 0.05). However, ovariectomy removed the female mice's protection against gaining weight, and in fact, ovariectomized female mice patterned like male mice in their susceptibility to weight gain (p > 0.05). Within male and female mice, after week 10 the body weights were significantly different among all groups (CR vs. LF vs. HF), before then only the CR mice had significantly lower body weights than LF and HF mice at week 5 (p < 0.05). For female vs. male mice (CR male vs. CR female, LF male vs. LF female, HF male vs. HF female) values were significant different at all time points respectively (p < 0.005). However, values were not significantly different between male and OVX female mice (p > 0.005). Percent body fat was determined by DEXA. Abbreviations: 30% calorie-restricted diet (CR), 5% low-fat diet (LF), 35% high-fat diet (HF). **B**, shows the percent body fat levels in male, female, and OVX-female C57BL/6 mice. Body fat levels between male and female mice were significantly different, with male mice having more body fat in all three diet categories than female mice (p < 0.05). Body fat levels in ovariectomized female mice were almost identical to those of male mice (within each category, male CR vs. CR OVX-female).

Fig 1A shows that all three male groups had greater propensity to gain weight and become obese than female mice (p < 0.05). Moreover, male mice consuming the LF diet developed body weights similar to female mice consuming the HF diet (p > 0.05). Differences in body weight between male and female mice were also reflected in differences in body fat levels (Fig 1B), with male mice having more body fat in all three diet categories than female mice (p < 0.05). To determine the effect of ovarian hormones on susceptibility to obesity in female mice, ovaries were surgically removed from 45 additional female mice, then divided into groups of 15 mice and given the three diets described above. Fig 1A shows that ovariectomized female mice resemble the male mice in their susceptibility to weight gain. Results also show that body fat levels in ovariectomized female mice were almost identical to those of male mice (Fig 1B). With respect to lean mass, values tended to be lower in male and OVX-female mice and higher in female mice. Lean body mass for male mice was (percent ± SE): CR 73 ± 1.0, LF 59 ± 1.9, and HF 42 ± 1.6; for female mice: CR 77 ± 1.4, LF 69 ± 1.7, and HF 56 ± 1.5; and for OVX-female mice: CR 71 ± 1.2, LF 55 ± 2.3, and HF 38 ± 3.6.

## Discussion

Review of the literature shows that no studies have made a direct comparison in susceptibility to body weight gain between males, females, and ovariectomized females consuming various diet regimens. Our studies show that male mice are more susceptible to obesity than female mice, and that ovariectomy eliminates the protection of female mice from becoming obese when exposed to high-fat diets. Furthermore, our results show that when female and OVX female mice consumed a high fat diet they both gain weight; however, OVX females gained more weight, specifically in the form of adipose tissue. We propose that ovarian hormones may protect females from diseases such as cancer by regulating aspects of metabolism and body composition. Evidence from animal studies supports this notion. Naugler et al. showed that male mice were more susceptible than females to liver cancer, the difference in susceptibility was eliminated by ovariectomy; and the addition of estrogen to ovariectomized female mice retrieved the protection against liver cancer [7]. Moreover, we showed that male mice have a higher susceptibility of becoming insulin resistant and developing tumors than female mice, and that ovariectomy removed the protection in female mice from becoming insulin resistant and developing tumors [3]. A limitation of our studies is that we did not include weight-matched male mice to the body weight of female mice. However, to include weight-matched mice, they would have to be forced to drop weight by interventions such as calorie restriction. Our unpublished observations are that calorie restricted mice over-consumed food after they are food-restricted; thus, it is possible that the variable used to generate weight-matched mice can become a confounder. However, the initial body weight of ovariectomized female mice was similar to male mice, and their weight gain pattern was similar to male mice, suggesting that ovarian hormones indeed provide protection against weight gain. Also, we did not distinguish between subcutaneous and visceral fat deposits in the different experimental groups; however, in our future studies we plan to further investigate the fat distribution among the experimental groups.

Mouse models of obesity have helped identify new targets for the treatment of diabetes [8,9]. The model presented here may allow the identification of key signaling pathways or genes that may explain the different susceptibility to obesity between males and females. As for the relevance of this model to human biology, previously, we showed that the CR, LF and HF diets induced similar body fat phenotypes in female mice, to those found in women considered lean (BMI less than 25), overweight (BMI between 25 and 30), and obese (BMI higher than 30), respectively [5]. Thus, with respect to body fat levels our diets are able to induce body fat levels (to some extent) to those found in humans. Moreover, it is possible that estrogen metabolism is different in mice and humans; and thus, key findings using this model would need to be verified in human samples.

Obesity is associated with an increased risk for a variety of mortality leading diseases including hypertension, cardiovascular disease and cancer [10-12]. Adult men are more likely to die from cancer and cardiovascular disease (CVD) than women [11,12]. Our hypothesis is that the difference in susceptibility to diseases such as cancer and CVD between males and females is due in part to fundamental physiological differences, including those that influence susceptibility to body weight gain. Furthermore, we propose that understanding metabolic differences between males and females may lead to the discovery of better treatments and preventive strategies for chronic diseases such as cancer.

## Competing interests

The authors declare that they have no competing interests.

## Authors' contributions

JH, RRS, AEH, participated in data collection, and data analysis, RES participated in manuscript preparation and editing, NPN participated in study design, data analysis and manuscript preparation. All authors read and approved the final manuscript.
